# NGS-diagnosed HBV encephalitis with isolated left parietal lobe involvement: a case report

**DOI:** 10.1186/s12985-026-03253-8

**Published:** 2026-07-16

**Authors:** Zihan Zhou, Zhangning Zhou, Longqi Zhang

**Affiliations:** 1 Department of Geriatric, Huzhou Central Hospital, Affiliated Central Hospital of Huzhou University, Huzhou, 313000 Zhejiang China; 2 Huzhou Central Hospital, Fifth School of Clinical Medicine of Zhejiang Chinese Medical University, Huzhou, 313000 Zhejiang China

**Keywords:** Viral Encephalitis, Hepatitis B virus, Cerebrospinal Fluid, Next-generation Sequencing

## Abstract

**Background:**

Viral encephalitis refers to the inflammation of the brain parenchyma caused by viral infection, usually manifested as fever, unconsciousness, seizures, and meningeal irritation symptoms. The non-specific clinical manifestations, combined with incomplete medical history, often lead to missed diagnoses and inappropriate treatments.

**Case presentation:**

We present a case of Hepatitis B virus (HBV) encephalitis, manifesting as isolated left parietal lobe involvement. The patient is a chronic hepatitis B virus carrier who did not receive antiviral therapy. Upon onset, the main symptoms were unconsciousness, fever, and seizures. He was diagnosed with HBV encephalitis thru next-generation sequencing (NGS) testing of the cerebrospinal fluid (CSF). After antiviral treatment with entecavir and general symptomatic treatment, the patient’s clinical symptoms disappeared, and a follow-up brain MRI showed that the lesion had almost completely resolved.

**Conclusions:**

Central nervous system (CNS) infections caused by HBV are incredibly uncommon in clinical practice. This case highlights that the clinical manifestations of HBV encephalitis are nonspecific and prone to being missed, while neuroimaging often reveals an isolated lesion. Early application of NGS for accurate pathogen diagnosis is crucial to reduce misdiagnosis.

## Background

Hepatitis B virus (HBV) infection affects approximately 296 million people worldwide and is a leading cause of liver disease [1]. While HBV is predominantly hepatotropic, a growing number of case reports describe extrahepatic manifestations, including neurological involvement such as polyradiculoneuritis [1, 2]. Most HBV-related neurological syndromes are secondary to hepatic encephalopathy or immune-mediated mechanisms, leading to low clinical suspicion and high misdiagnosis rates of pure HBV encephalitis. The first confirmed case of HBV encephalitis without coinfection was reported recently with isolated caudate nucleus involvement [3]. Here, we present a second case of pure HBV encephalitis diagnosed by cerebrospinal fluid (CSF) next-generation sequencing (NGS), presenting with isolated left parietal lobe involvement, to clarify its clinical and radiological features and diagnostic value.

## Case presentation

A 38-year-old man was admitted with a 3-hour history of unconsciousness, accompanied by fever, irritability, aphasia, and focal seizures. He had no underlying immunocompromised condition or history of immunosuppressive therapy. His only underlying condition was chronic hepatitis B, for which he had not received antiviral therapy.

On examination, he had a fever (38.1℃) with impaired calculation, disorientation and reduced interaction. He had nuchal rigidity and Babinski’s sign, normal muscle power, elevated muscle tone, and symmetrical forehead wrinkles and nasolabial folds. The remainder of the neurological examination could not be performed, and no signs of chronic liver disease were observed.

Blood tests showed positive for hepatitis B surface antigen (HBsAg), hepatitis B e-antibody (HBeAb) and hepatitis B core antibody (HBcAb), HBV DNA 8.81 × 10⁶ IU/mL, alanine aminotransferase (ALT) 105.2 U/L, aspartate aminotransferase (AST) 74.1 U/L, serum ammonia 40.3 µmol/L, albumin (ALB) 31.0 g/L, total bilirubin (TBil) 25.9 µmol/L, prothrombin time (PT) 14.1 s, international normalized ratio (INR) 1.25, platelet count 44 × 10⁹ g/L. Tests for hepatitis A virus, hepatitis C virus, hepatitis D virus, hepatitis E virus, autoimmune hepatitis, inflammatory markers, and serum electrolytes were unremarkable. Contrast-enhanced MRI of the abdomen revealed signs of cirrhosis, splenomegaly, and massive ascites. CSF analysis showed an opening pressure of 300 mmH₂O, nucleated cell count 0.119 × 10⁹/L (87.4% being mononuclear cells), protein 1642.0 mg/L, glucose 6.58 mmol/L, and chloride 126.0 mmol/L. Cultures of CSF for general bacteria and fungi were negative, and no Cryptococcus was detected. CSF NGS identified 3 HBV sequences (98.55% relative abundance) and 14 Enterococcus avium sequences (0.41% relative abundance). CSF was positive for HBsAg, HBeAb, and HBcAb, with detectable HBV DNA (3.47 × 10² IU/mL). Autoimmune encephalitis antibody panels (including anti-NMDAR、AMPAR1、AMPAR2、LGI1、CASPR2、GABABR、DPPX、IgLON5、GlyR1、D2R、GAD65、mGluR5、mGluR1、GABAARα1、GABAARβ3、Neurexin3α、MOG、GFAP) and HIV/syphilis serologies were negative.

**Fig. 1:**
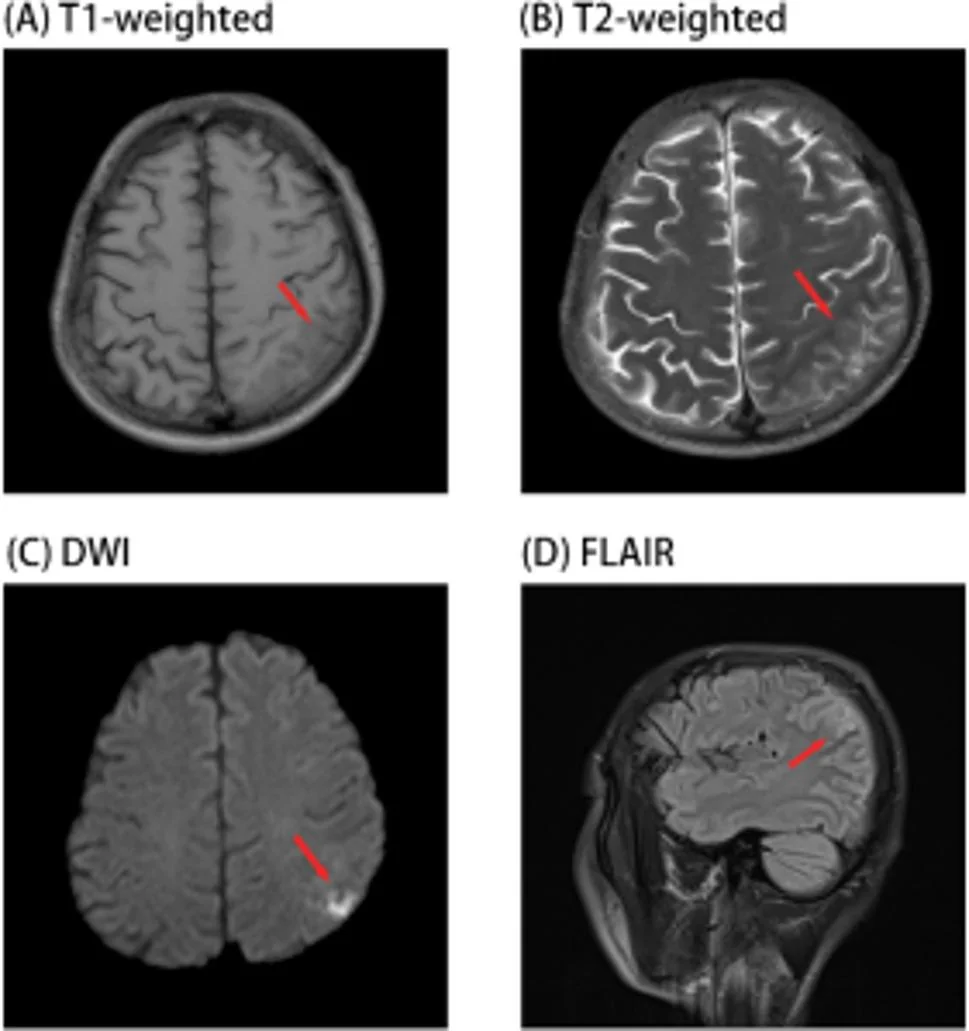
Brain Magnetic Resonance Imaging (MRI) Findings. Brain MRI showed a left parietal lobe hypointense lesion (arrow) on T1-weighted sequence (**A**) and hyperintense lesions (arrow) on T2-weighted (**B**), diffusion-weighted imaging (DWI) (**C**), and fluid-attenuated inversion recovery (FLAIR) (**D**) sequences before treatment

**Fig. 2:**
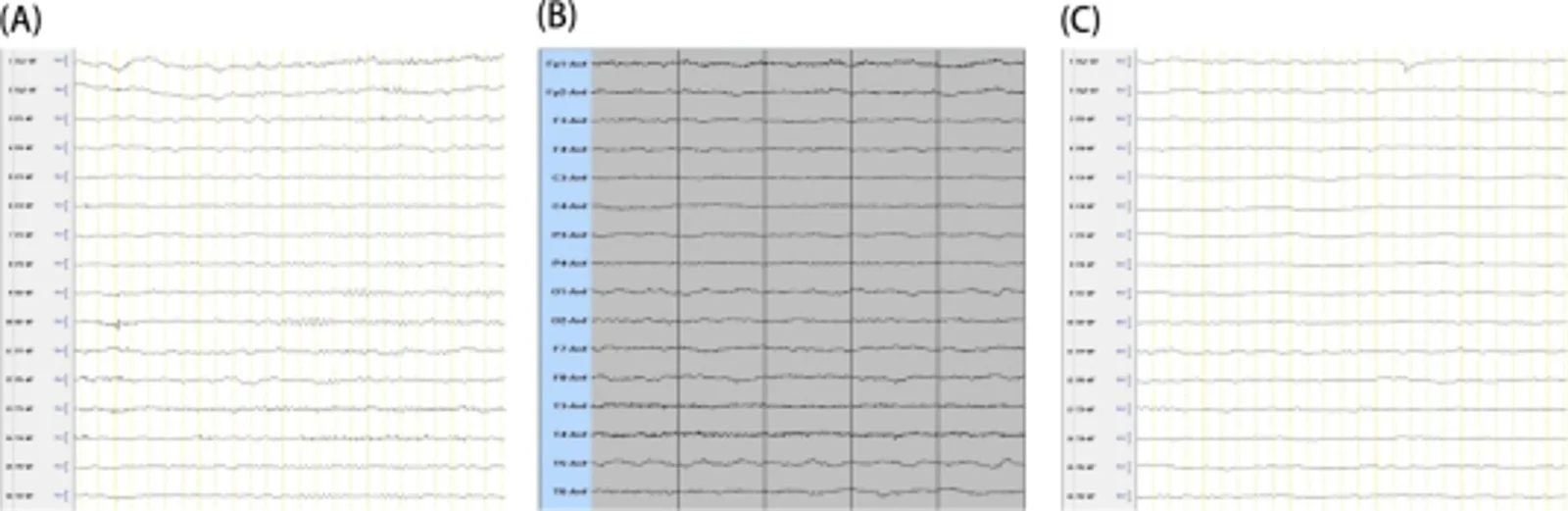
Electroencephalography (EEG) findings. The EEG on the second day after admission showed diffuse slow waves (**A**), the EEG on the seventh day after admission (following a focal epileptiform seizure) showed focal slow waves in the left parieto-occipito-temporal region (**B**), and the EEG appeared normal after 1.5 months of entecavir therapy (**C**)

Brain magnetic resonance imaging (MRI) revealed left parietal lobe hypointensity on T1-weighted and hyperintensity on T2-weighted/diffusion-weighted imaging (DWI)/fluid-attenuated inversion recovery (FLAIR) (Fig. 1). The electroencephalography (EEG) on the second day after admission showed diffuse slow waves (Fig. 2A), whilst the EEG on the seventh day after admission (following focal seizures) showed focal slow waves in the left parieto-occipital-temporal region (Fig. 2B).

**Fig. 3:**
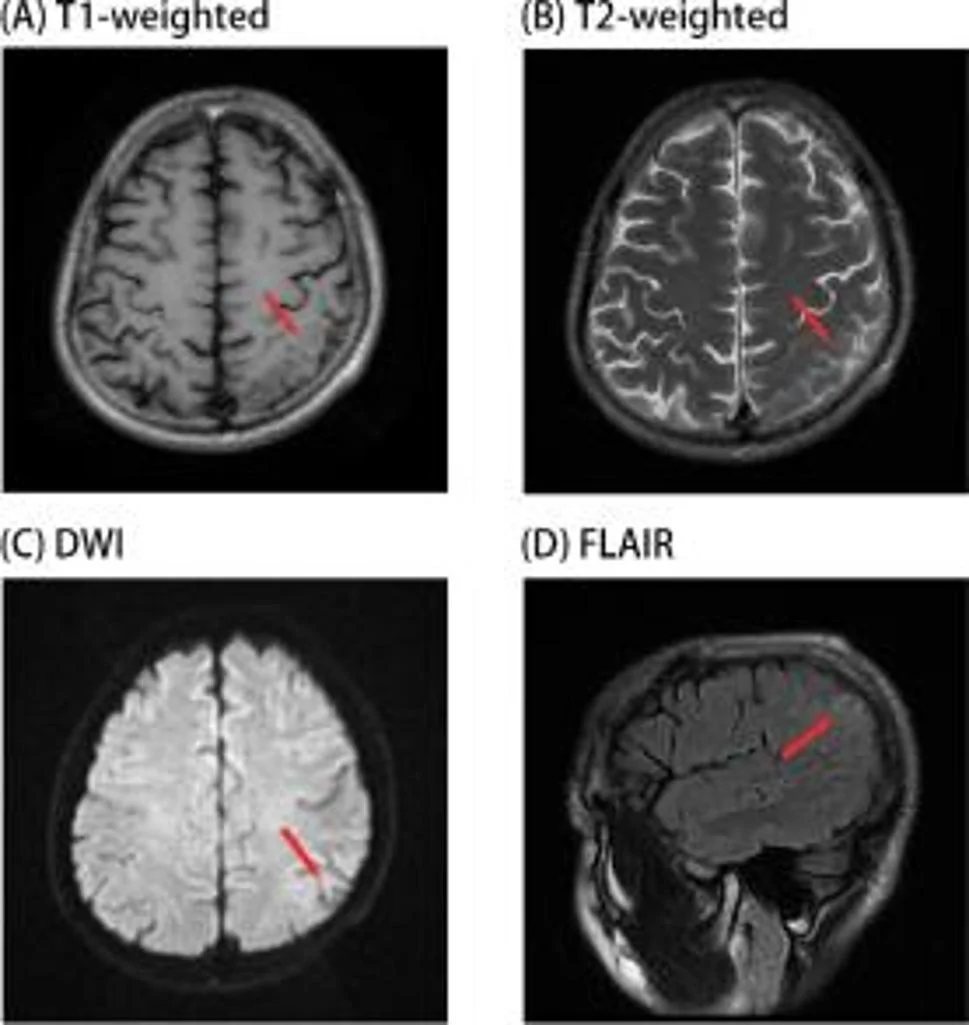
Follow-up Brain Magnetic Resonance Imaging (MRI) Findings. Follow-up brain MRI (**A**/**B**/**C**/**D**) after 1.5 months of entecavir therapy shows near-complete resolution of the lesion (arrow)

After admission, empirical treatment with antibiotics, antivirals, and intracranial pressure reduction was administered. Following the auxiliary examination results, the patient was diagnosed with HBV encephalitis, and additional antiviral therapy with entecavir was administered. At 1.5-month follow-up, he had no recurrence of seizures or encephalopathy. Follow-up serum HBV DNA levels had decreased (5.18 × 10² IU/mL), and liver function had improved (ALT 55.0 U/L, AST 48.0 U/L, ALB 42.7 g/L, TBil 41.3 µmol/L, PT 12.7 s, INR 1.13). CSF pressure was reduced (130 mmH₂O), and protein content was decreased (396.0 mg/L). Repeat NGS of CSF was negative for HBV, and HBV DNA was not detected. EEG revealed no abnormalities (Fig. 2C), and brain MRI showed near-complete resolution of the parietal lesion (Fig. 3).

### Discussion and conclusions

The diagnosis of viral encephalitis relies on the identification of a pathogen in the central nervous system (CNS), often amidst a wide differential diagnosis that includes autoimmune causes and other infections [4]. Pure HBV encephalitis without coinfection is an extremely rare clinical entity with limited reported cases. We report only the second case of HBV encephalitis, providing independent validation for this emerging clinical entity. The definitive diagnosis was achieved through the application of NGS on CSF, which detected HBV sequence, a finding supported by the concurrent detection of HBV markers and DNA in the CSF. This case underscores the utility of NGS as a hypothesis-free tool for diagnosing unusual CNS infections, particularly when conventional tests are unrevealing.

HBV is a widely distributed DNA virus that primarily acts on the liver, and neurological involvement is a rare extrahepatic manifestation [5, 6]. The true incidence of HBV encephalitis is unknown, with this being only the second well-documented case. The pathogenesis of HBV-induced encephalitis is unknown, which may be through direct viral invasion or immune mediation. The fact that our patient had a high viral load at onset suggests that high levels of viral replication may be a predisposing factor. The rapid radiological and clinical response to antiviral therapy further strengthens the association between the lesion and active HBV infection. Further research is needed to understand the pathogenesis of HBV encephalitis to better manage hepatitis B patients and prevent serious neurological complications.

As with encephalitis caused by other pathogens, the patient in this case primarily presented with consciousness disorders and fever, lacking specific manifestations, which greatly increased the difficulty of clinical diagnosis and treatment. Unlike the first case of HBV encephalitis reported by Zheng et al. [3], this is the first patient to present with an isolated lesion in the left parietal lobe on imaging. This finding further expands the radiological spectrum of CNS HBV infection, while also indicating that HBV’s neurotropism doesn’t have a specific brain region preference, but usually presents as isolated involvement. This isolated, non-anatomical distribution helps to distinguish it from other encephalopathies, such as: (1) herpes simplex virus encephalitis, which primarily affects the temporal lobe; [7] (2) anti-LGI1 autoimmune encephalitis (AE), anti-CASPR2 AE, anti-GABABR AE and anti-AMPAR AE, which typically affect the medial temporal lobe, including the hippocampus, amygdala, hypothalamus, cingulate gyrus and limbic cortex, presenting as limbic encephalitis; anti-CV2/CRMP5 AE usually affects extra-limbic structures, presenting as striatal encephalitis; [8] (3) vascular events, with lesions predominantly distributed along blood vessels. [3]

There are no established guidelines for treating HBV encephalitis. Management principles are extrapolated from the treatment of chronic hepatitis B. Our patient was initiated on the nucleoside analogue entecavir, which has high antiviral efficacy and a high barrier to resistance. The subsequent clinical recovery, clearance of HBV from the CSF, and resolution of the brain lesion suggest that early and potent antiviral therapy is beneficial. This parallels the management strategy in the first reported case, where tenofovir was used [3]. Unlike the complex antifungal regimens often required for infections like cerebral phaeohyphomycosis [9], the management here is more straightforward, focusing on single-agent antiviral suppression. However, the optimal duration of therapy is unknown, and long-term suppression may be prudent given the chronic nature of the underlying HBV infection.

We report the second case of pure HBV encephalitis without coinfection diagnosed by CSF NGS, and this is the first case presenting as isolated left parietal lobe involvement. This case expands the neuroimaging spectrum of intracranial HBV infection. Given its non-specific presentation and clinical rarity, HBV encephalitis is easily misdiagnosed. Early NGS testing of CSF can help identify the pathogens associated with infection-related encephalitis, guiding clinical treatment and improving clinical outcomes. In clinical practice, when encountering hepatitis B patients with neurological symptoms or isolated lesions on brain MRI, one should be vigilant for HBV encephalitis, immediately conduct CSF NGS testing, and start anti-HBV treatment as soon as possible.

## Data Availability

The datasets used and/or analysed during the current study are available from the corresponding author on reasonable request.

## Abbreviations

HBV: Hepatitis B virus
CSF: Cerebrospinal fluid
NGS: Next-generation sequencing
MRI: Magnetic resonance imaging
DWI: Diffusion weighted imaging
FLAIR: Fluid-attenuated inversion recovery
EEG: Electroencephalography
HBsAg: Hepatitis B surface antigen
HbeAb: Hepatitis B e antibody
HBcAb: Hepatitis B core antibody
ALT: Alanine aminotransferase
AST: Aspartate aminotransferase
ALB: Albumin
TBil: Total bilirubin
PT: Prothrombin time
INR: International normalized ratio
CNS: Central nervous system
